# 

**Published:** 1837

**Authors:** 


### CONTENTS OF VOL. XI.

### NO. XLI.

### ESSAYS AND CASES.

Page.

ART. I.—HOSPITAL REPORTS.—*Cases treated in the Cincinnati Hospital, with Clinical Observations:* Recorded by William J. Barbee, M. D., and Edward Kimball, House Surgeons. Arranged by the Editorial Committee. 9

II.—CLINICAL INTRODUCTORY.—*The Indications of Cure in Diseases: An Introductory Lecture, to a Course of Clinical Instruction in the Cincinnati Hospital.* By John P. Harrison, M. D. 34

III.—MORBID ANATOMY OF THE LIVER.—*Outlines of the Morbid Anatomy of the Liver.* By. S. D. Gross, M. D. 43

REVIEWS AND BIBLIOGRAPHICAL NOTICES.

IV.—BLOOD LETTING.—*An account of the Curative Effects of the abstraction of blood; with the rules for employing both local and general blood-letting in the treatment of diseases.* By James Wardrop, M. D. 71

V.—HOMŒPATHIA.—*Homœpathic Medicine. Illustrating its superiority over the other medical doctrines, with an account of the regimen to be followed during the treatment of diseases.* By M. Croserio, M. D. 88

VI.—DISEASES OF THE UTERUS.—*A Treatise on the Functional and Organic Diseases of the Uterus.* From the French of M. Duparcque, M. D. 92

---

MISCELLANEOUS INTELLIGENCE.—ANALECTIC.

ART. 1.—Some Experiments and Observations on tying the carotid and vertebral arteries, and the pneumo gastric, perenic and sympathetic nerves. By Sir Astley Cooper, Bart. 105

2.—Physiological action of Iodine and Hydriodic Acid. By Andrew Buchanan. 116

3. Iodide of Potassium. 120

4. Results of Experiments with Creosote. 121

5. Iodide of Starch. 122

6. Dropsy following Scarlet Fever. By J. Stark, M. D. 123

7. Efficacy of Chlorine in Scarlatina. 123

8. New method of reducing luxations of the Humerus. By M. C. Gerard, M. D. 124

9. New treatment of Strictures of the Urethra. By M. Jobert. 125

10. New treatment of Onychia Maligna. By C. Ray, Esq., Surgeon. 125

11. On Binding the Abdomen of Lying-in Women. By Hugh Ley, M. D. 125

Page.
12. Nitrate of Silver in Diseased Stomach. 126
13. Some observations on the use of the Hydriodate of Potash as a remedy for Venereal and Cachectic Symptoms. 127

ANALYTICAL.

Art. I.—Graduates of the University of Pennsylvania. 138
II.—Medical Proceedings in the State of Connecticut. 141
III.—Medical School of Fairfield. 144
IV.—Private Anatomical School, at Auburn, New-York, 145
V.—The Willoughby Medical College. 148
VI.—Medical Department of Transylvania University. 150
VII.—Acclimation. Temperance in the South. Medical College of Louisiana. 152
VIII.—The American Cyclopædia of Practical Medicine and Surgery. 155
IX.—Bell's and Dunglison's Medical Libraries. 156
X.—Philosophy of Medical Investigation. 156

ORIGINAL.

Art. I.—New Prospectus of the Western Journal of the Medical and Physical Sciences. 159
II.—Professor Parker's Letter from Paris. 160
III.—Re-organization of the Medical Department of Transylvania University. 162
IV.—Louisville Medical Institute. 163
V.—The Medical College of Ohio. 163
VI.—The College of Surgeons and Physicians of the University of the State of New-York. 163
VII.—Hamilton County Medical Association. 165
VIII.—Medical and Physical Works in the Library of the Lane Theological Seminary. 166
IX.—Medical Department of the Cincinnati College. 168
Bibliographical Record, or Quarterly List of New Publications in Medicine, Surgery, and the Associate Sciences. 169
Charter and Recognition of the Cincinnati College. 173

---

NO. XLII.

ESSAYS AND CASES.

Art. I.—Hospital Reports. 177
II.—Case-Book Extracts.—*Several Short Cases of Disease,* extracted from the Note Book of Dr. A. B. Price, 189
III.—Spinal Endermic Medication.—*Cases illustrating the efficacy of medicinal application to the region of the Spine.* By Dr. G. J. Cowles. 192

REVIEWS AND BIBLIOGRAPHICAL NOTICES.

IV.—General Therapeutics. By Robley Dunglison, M. D. 199
V.—Diseases of the Eye. By S. Littell, M. D. 225
VI.—Diseases of the Uterus. By M. Duparcque, M. D. 249

### MISCELLANEOUS INTELLIGENCE.—ANALECTIC.

*Page.*

1. Physiology. Experiments on the mechanism of the motion or beating of the Arteries. By M. Flourens. 177*
2. The Saliva. As indicative of the state of the stomach in health and disease. 179
3. Experimental Researches in the physiology of the human voice. By John Bishop, Esq. 180
4. On the safety-valve of the right ventricle of the heart in Man; and on the gradations of the same apparatus in the Mammalia and Birds. By J. W. King, Esq. 181
5. On the Brain of the Negro compared with that of the European and Ourang-outang. By F. Tiedemann, M. D. 181
6. The Factory System and Factory Statistics. 182
7. Special Anatomy. On the Elementary Structure of the Muscular Fibre of Animal and Organic Life. 187
8. On the connexion of the Anterior Columns of the Spinal Cord with the Cerebellum. 188
9. On the Functions of the 5th, 6th and 7th pairs of Nerves. 189
10. On the Functions of the Medulla Oblongata and Medulla Spinalis, and on the Excito-motory system of Nerves. 189
11. On the importance of Percussion in the Diagnosis of Ascites and encysted Ovarian Dropsy. 190
12. Clinical researches on the influence of certain medicines on the functions of the heart. By H. C. Lombard. 191
13. Physiological effects of Tannin. 192
14. Therapeutic application of Indigo. 193
15. Physiological operation of Indigo. 194
16. Observations on Jaundice; more particularly on that form of the disease which accompanies the diffused inflammation of the substance of the liver. By Richard Bright, M. D. 195
17. Pins taken from the Body. 300
18. Treatment of Hydrocele by injections of Iodine. 301
19. On Pessaries, and the radical cure of Prolapsus Vaginæ et Uteri. By Prof. Dieffenbach. 302
20. Treatment of some forms of acute Ophthalmia, by the application of successive Blisters upon the cutaneous surface of the Eyelids. 303
21. Prof. Rust's method of operating for Cataract by Reclination. 304
22. On the Pneumonia of old people. By Hourmann and Dechambre. 306
23. Morbid Anatomy of Typhus Fever in Paris, Geneva, Dublin, Glasgow, Liverpool, Manchester and London. By Dr. Lombard, of Geneva. 311
24. Contagious Typhus. By Dr. Perry. 313
25. On the Motions and Sounds of the Heart. By Drs. Williams, Todd and Clendinning. 314

---

*By mistake of the compositor, the paging of signatures 13, 14, and 15 is wrong. This page should have been 273.

Page.
26. On the Chemistry of the Digestive Organs. By R. D. Thomson, M. D. 315
27. Physical signs of the height of the Diaphragm in the Chest. By Edwin Harrison, M. D. 315
28. On the Causes of Resonance in Percussion. By John Clendinning, M. D, 316
29. On the Morbid appearance in death from drowning. By W. Ogeston, M. D., of Aberdeen. 316
30. On the present state of Medicine in Italy. 317
31. Scientific discoveries in Germany. By Prof. Weber. 319

ANALYTICAL.

I.—Physiological Observations on the Pulsations of the Heart, and its diurnal revolutions and excitability. By Dr. Knox, of Edinburgh. 320
II.—Large doses of Sulphate of Quinine, in Malignant Intermittents. By Dr. May. 321
III.—Milk-Sickness. By Dr. Smith. 322
IV.—Microscopic Insects, apparently developed from inorganic matter, by Galvanism. 325
V.—Morrison and Brandreth's Pills. 327
VI.—The Graham Journal of Health and Longevity. 329
VII.—Medical Journals and Medical Schools. 331
VIII.—*De Abortu.* 332

ORIGINAL.

I. Our Institution, p. 335. II. Filling up of the Louisville Medical Institute, 336. III. Reorganization of the Medical College of Ohio, 337. IV. Medical Convention of Ohio, 337. V. Voluntary Medical Societies in Kentucky, 339. VI. Fœtal Monster; by Dr. Prout, 340. VII. Successful Tracheotomy; by Dr. Mount, 341. VIII. Cow-pox and Measles at the same time, in the same patient, 342. IX. Variantia Rerum, 343.

---

NO. XLIII.

ESSAYS AND CASES.

Art. I.—Hospital Reports. 345
II.—Hepatic Abscess opening externally.—*Discharge of Biliary Calculi—Recovery.* Communicated by Dr. G. C. Starbuck, of Rochester, Ohio. 369
III.—Elm Bark in Surgery.—*An Account of the use of the Bark of the Slippery Elm Tree (ulmus fulva), for Bougies, Tents, Catheters, and similar purposes in Surgery.* By W. A. McDowell, M. D., of Fincastle, Virginia. 371
IV.—Death from Ulceration of the Appendix Vermiformis. By Wolcott Richards, M. D., of Cincinnati. 376
V.—Spinal Endermic Medication. (Continued from last No.) 379

Page.

### REVIEWS AND BIBLIOGRAPHICAL NOTICES.

VI.—Diseases of the Rectum and Anus.—*A Treatise on the Malformations, Injuries, and Diseases of the Rectum and Anus.* By George Bushe, M. D., formerly Professor of Anatomy and Physiology. 385

VII.—Animal Magnetism.—*Report of Dr. Franklin and other Commissioners, charged by the King of France with the examination of Animal Magnetism, as practised at Paris, &c.* 407

### MISCELLANEOUS INTELLIGENCE.—analectic.

1. Epidemic Influenza, 441. 2. Influenza in France, 441. 3. Debate in the Academy of Medicine of France relative to the Influenza, 445. 4. Influenza in France, 447. 5. do. in Belgium, 449. 6. do. at Copenhagen, 452. 7. On the Enlargement of the Spleen, after agues and other fevers, 454. 8. The Efficacy of Lobelia Inflata in Inflammations and Congestions of the Mucous Lining of the Bronchial Tubes; by Dr. Cartwright, 460. 9. On the Ciliary Motions in Man; by Dr. Von Siebold, 462. 10. On the Ciliary Motions in the Brain; by M. Purkinge, 462. 11. On the Permenent Immoveable Bandage; by Dr. Suetin, 463. 12. Chronic Catarrh of the Bladder, treated by Injections, by Devergie, 464. 13. On Pessaries, and the Radical Cure of Prolapsus Vaginæ et Uteri, 465. 14. On the use of the Plug in cases of Placenta Prævia, 466. 15. Two cases of Complete Recovery from Laceration of Vagina and Uterus, 466. 16. Section of the Tendo-Achillis, 467. 17. Medical Education in Berlin, 470. 18. The Medical Profession, 473. 19. Statistics of Hospitals, 475. 20. Use of Solid Nitrate of Silver in the Gonorrhœa of Women, 481. 21. Case of Recovery from the Insensibility of Intoxication, by the performance of Tracheotomy, 482. 22. Case of Recovery from the Influence of Opium, by Artificial Respiration, 483.

analytical.

1. Observations and Experiments on Softening, Erosion, and Perforation of the Stomach; by Dr. Imlach, 485. 2. Typhus Fever in Philadelphia in 1836; by Dr. Gerhard, 489.

Original.—1. Editorial Gossip, 497. 2. Obituary Notices, 502

Bibliographical Record, or Quarterly List of New Publications in Medicine, Surgery, and the Associate Sciences, 503. Appendix, embracing an account of the Proceedings of the Medical Convention of the State of Ohio, held at Columbus, January, 1838, 507.

---

### NO. XLIV.

### ESSAYS AND CASES.

Art. I.—Hospital Reports. 507

II.—Alterants.—*An Essay on Alterants.* By John P. Harrison, M. D. 515

Page.

III.—VAGINAL OBLITERATION.—*Two cases of the Obliteration of the Vagina, caused by ulceration.* By Thos. Carrall, M. D., of St. Clairsville, Ohio: 546

IV.—RECOVERY FROM A WOUND IN THE STOMACH. By Chas. B. New, M. D., of Rodney, Miss. 551

V.—TRACHEOTOMY IN TRONSILITIS.—*A case of Tronsilitis requiring, and successfully treated, by Tracheotomy.* By Dr. Jas. Lyman, of Austinsburg, Ohio. 552

VI.—ODONTALGIA.—*Observations on Toothache.* By S. P. Hullihen, of Wheeling, Va. 555

VII.—DEATH FROM EATING FRUIT.—*A case of Death apparently from eating unripe Apples, and the fruit of the Prunus Virginiana, or Wild Cherry.* By Dr. R. C. Mann, of Mount Pleasant, Hamilton county, Ohio, 562

VIII.—MINERAL SPRINGS OF VIRGINIA.—*Notices of the Blue Sulphur and Hot Springs of Virginia.* By one of the Editors. 565

REVIEWS AND BIBLIOGRAPHICAL NOTICES.

IX.—AMERICAN MEDICAL SCHOOLS.---*Report on the Organization and Administration of the Medical Schools of the U. S.* 572

X.---DISEASES OF THE LARYNX.---*A Practical Treatise on Laryngeal Phthisis, Chronic Laryngitis, and Affections of the Voice.* By A. Trousseau and H. Bellog. 579

---

MISCELLANEOUS INTELLIGENCE.---ANALECTIC.

1. Treatment of Paralysis, at Paris, with the Actual Cautery-- New mode of Blistering, p. 603. 2. On Sedative Poison, 606. 3. On the Human Voice, 617. 4. New Experiments of the Sense of Taste in Man, 621. 5. Severe Epileptic Convulsions cured by compressing the Carotid Artery, 623. 6. Poisoning by Cream of Tartar, *ib.* 7. Polypi in the Mentus Auditorus Externus, 625. 8. Intermittent Neuralgiæ, 628. 9. Case of Malformation of the Septum Narium, 630. 10. American Crania, 631. 11. Nitrate of Silver in immense doses, 634. 12. On Creosote as an external application, 635. 13. On the treatment of Ileus with Belladonna Clysters, 637. 14. On St. Vitus's Dance, 640. 15. On the first changes in the Ova of Mammifera---The mode of Origin of the Chorion, 642.

ANALYTICAL AND ORIGINAL.

1, To our Subscribers, 641. 2, Cincinnati College, 642. 3, Elements of Pathology, 642. 4, Medical Convention of Ohio, 643. 5, The Medical Examiner, 644. 6, Obituary Notices, 648. 7, Compensation of Physicians, 651. 8, Rotation in Office, 651. 9, Varicose Veins and Ulcers of the Legs, 652. 10, Geological Surveys of Ohio, Indiana and Michigan, 659. 11, Medical Schools of the Valley of the Mississippi, 662. 12, Bandage for the cure of Prolapsus Uteri, 663. 13, British Journals, 663. 14, To the Patrons of the Western Journal, 664.

EXTRA-LIMITES.—Art. I. Catalogue of the Medical Department of the Cincinnati College, 665. II. Reports on the Medical College of Ohio, 670. Bibliographical Record, 676.

